# The role of the striatum in social behavior

**DOI:** 10.3389/fnins.2013.00233

**Published:** 2013-12-10

**Authors:** Raymundo Báez-Mendoza, Wolfram Schultz

**Affiliations:** Department of Physiology, Development and Neuroscience, University of CambridgeCambridge, UK

**Keywords:** social interactions, social neurophysiology, agency, value, human, macaque, vole, rat

## Abstract

Where and how does the brain code reward during social behavior? Almost all elements of the brain's reward circuit are modulated during social behavior. The striatum in particular is activated by rewards in social situations. However, its role in social behavior is still poorly understood. Here, we attempt to review its participation in social behaviors of different species ranging from voles to humans. Human fMRI experiments show that the striatum is reliably active in relation to others' rewards, to reward inequity and also while learning about social agents. Social contact and rearing conditions have long-lasting effects on behavior, striatal anatomy and physiology in rodents and primates. The striatum also plays a critical role in pair-bond formation and maintenance in monogamous voles. We review recent findings from single neuron recordings showing that the striatum contains cells that link own reward to self or others' actions. These signals might be used to solve the agency-credit assignment problem: the question of whose action was responsible for the reward. Activity in the striatum has been hypothesized to integrate actions with rewards. The picture that emerges from this review is that the striatum is a general-purpose subcortical region capable of integrating social information into coding of social action and reward.

## Introduction

The striatum is necessary for voluntary motor control. Research on its role in movement planning and execution uncovered its participation in cognition and reward processes. Rigorous experimentation demanded social isolation to properly study this neuronal circuit. However, action, rewards and cognition also occur in the company of conspecifics, in a social context. Social behaviors, those behaviors that occur in a social context, place an extra demand on cognition since others' behaviors are difficult to predict and they affect our own behavior. Therefore, to understand the properties of the striatum it is important to study it while the organism engages in social behavior. Recent studies highlight this brain structure during different social behaviors. Among these studies, we found that the striatum contains neurons that signal the social action that will result in own reward. We place these new findings within the context of previous findings on the known role of this area in movement and reward coding in the brain. The question that guides the review is as follows: “does the striatum serve a social function?” We conclude that the striatum is a general-purpose subcortical region capable of integrating and reflecting social information into its better known non-social functions.

## Anatomy and neurophysiology of the striatum

The striatum is the input module to the basal ganglia, a neuronal circuit necessary for voluntary movement control (Hikosaka et al., 2000). The striatum is composed of three nuclei: caudate, putamen, and ventral striatum. The latter contains the nucleus accumbens (NAcc). The caudate and putamen/ventral striatum are separated by the internal capsule, a white matter tract between brain cortex and brainstem.

**Figure 1:**
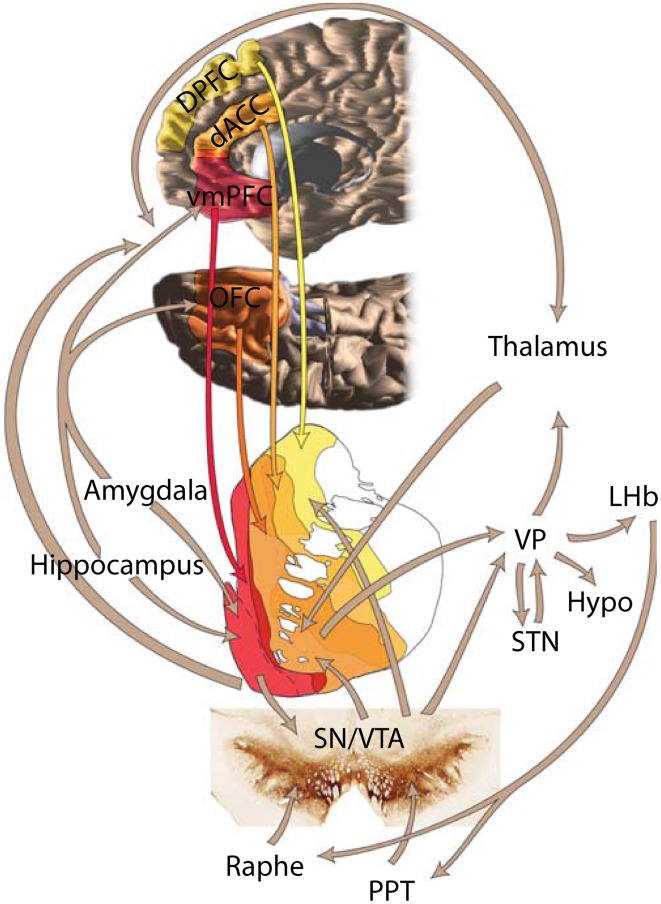
**Depiction of the brain's reward circuit highlighting the role of the striatum and its anatomical connections**. Abbreviations: dACC, dorsal anterior cingulate cortex; DPFC, dorsal prefrontal cortex; vmPFC, ventromedial prefrontal cortex; VP, ventral pallidum; LHb, lateral habenula; Hypo, hypothalamus; STN, subthalamic nucleus; SN, substantia nigra; VTA, ventral tegmental area; PPT, pedunculopontine tegmentum. Based on Haber and Knutson (2010), reproduced with permission.

Striatal afferents arrive from three major sources: cortex, midbrain and thalamus (Selemon and Goldman-Rakic, 1985; Haber, 2003). The cortical input from temporal, parietal and frontal is mostly ipsilateral (Künzle, 1975; Vanhoesen et al., 1981) and topographically arranged in the medio-lateral and dorsal-ventral axes (Selemon and Goldman-Rakic, 1985; Haber, 2003; Haber and Knutson, 2010). The striatum receives inputs from all elements of the reward circuit (Figure 1, reviewed in Haber and Knutson, 2010): from striato-nigral midbrain cells (Beckstead et al., 1979), amygdala (Russchen et al., 1985; Fudge et al., 2002), orbitofrontal cortex (OFC) (Haber et al., 2006), and anterior cingulate cortex (ACC) (Selemon and Goldman-Rakic, 1985; Calzavara et al., 2007).

The striatum has two main efferent pathways. The direct pathway is formed by axons of medium spiny neuron (MSN) expressing D1 receptors which mainly project to GABAergic neurons in the substantia nigra pars reticulata (SNr) (Parent et al., 1984; Gerfen et al., 1990; Kawaguchi et al., 1990; Chuhma et al., 2011). MSN that express D2 receptors mostly target the external segment of the globus pallidus (GPe) and form the indirect pathway (Parent et al., 1984; Gerfen et al., 1990; Kawaguchi et al., 1990; Chuhma et al., 2011). GABAeric neurons in GPe project to SNr and the internal segment of the globus pallidus (GPi) (Parent and Hazrati, 1995; Wilson, 1998). The SNr and GPi are the output nuclei of the basal ganglia.

The principal cell type in the striatum is the MSN (Wilson, 1998; Tepper and Bolam, 2004). These neurons release γ-amino butyric acid (GABA) at their synaptic terminals (Wilson, 1998). The striatum contains many other cell types besides MSN, including cholinergic and fast-firing GABAergic interneurons (Tepper and Bolam, 2004). Cholinergic interneuron activity has a relationship to reward-predicting stimuli and reward and punishment (Apicella et al., 1991b; Ravel et al., 2003). These firing properties suggest that these neurons may play a role in learning (Schulz and Reynolds, 2013). Fast-firing interneurons are also involved in reward prediction error coding (Stalnaker et al., 2012). However, for brevity we will limit this review to MSN and refer to them as striatal neurons. Functionally, striatal neurons show motor and reward responses (Hikosaka et al., 2000). Functional and anatomical evidence led to the hypothesis that striatal activity forms a “limbic-motor” interface (Mogenson et al., 1980). Neurons in the striatum integrate information about expected reward with motor information to guide behavior (Hollerman et al., 1998; Hikosaka et al., 2000; Schultz, 2000; Schultz and Dickinson, 2000; Goldstein et al., 2012). We review MSN neurophysiological responses to action and reward in the next section.

### Striatum neurophysiology: action and reward

**Figure 2:**
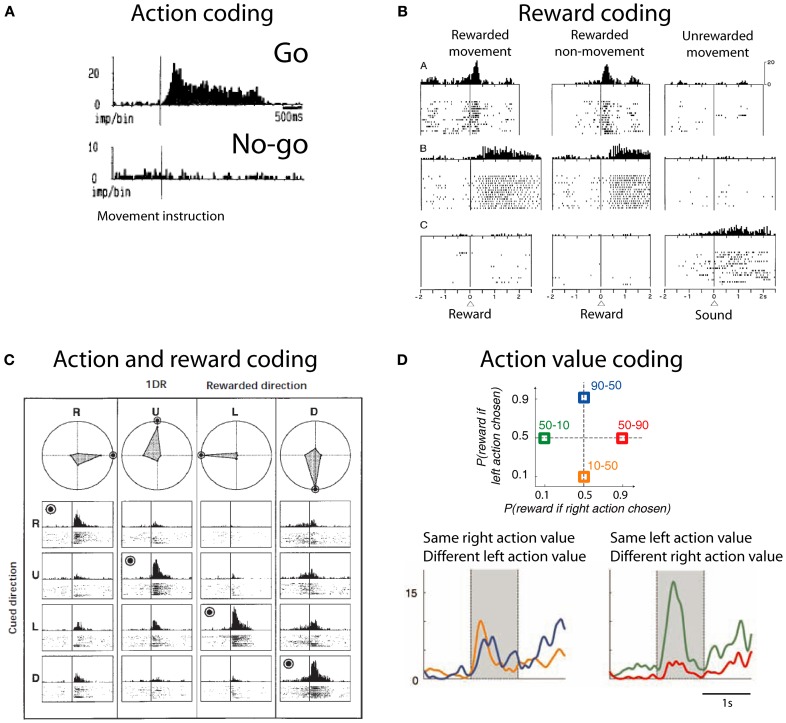
**Action and reward coding by striatal neurons. (A)** Example striatal neuron active before movement (go) and silent before no-movement (no-go). Based on Schultz and Romo (1988), reproduced with permission. **(B)** Example striatal neurons coding reward. First row depicts a neuron with phasic active after juice reward delivery independent of the action to obtain reward. Second row depicts a neuron with tonic activity after juice reward delivery. Third row shows a neuron with tonic activity after no reward is delivered. Based on Hollerman et al. (1998), reproduced with permission. **(C)** Example caudate neuron coding the conjunction of action and reward. This neuron is active during the presentation of a cue indicating the saccade necessary to complete the trial if the trial will be rewarded (rewarded direction is highlighted by a bulls eye). R, right; U, up; L, left; D, down. Polar plots show the average response for each cue and direction. Based on Kawagoe et al. (1998), reproduced with permission. **(D)** (Top) Depiction of the probability of larger rewards associated with left or right actions on each condition block. Colored numbers refer to the probability associated with left-right actions. (Bottom) Example striatal neuron coding right action value. Based on Samejima et al. (2005), reproduced with permission.

The striatum contains neuronal activity related to movements, rewards and the conjunction of both movement and reward. Striatal neurons show activity related to the preparation, initiation and execution of movements (Hollerman et al., 2000). These neurons are also active before overt goal-directed movements (Schultz and Romo, 1988; Romo et al., 1992; Figure 2A). Some of these neurons are exclusively active during self-initiated movements, whilst other neurons are only active during instructed trials, and some others do not discriminate between self-initiated and instructed movements. In addition to this, striatal neurons also show reward related activity. Neuronal activity in the striatum is modulated by reward expectation independent of the movement necessary to obtain it (Hikosaka et al., 1989b; Apicella et al., 1991a, 1992; Schultz et al., 1992). Striatal neurons that discharge after reward delivery do so in two main modes: phasic or tonic. Phasic responses usually have short latencies (<50 ms) and are relatively short lived—median duration: 500 ms (Apicella et al., 1991b; Hollerman et al., 1998; Lau and Glimcher, 2007; Figure 2B). By contrast, tonic responses have longer latencies and can last as long as the intertrial interval, i.e., up to 3 s (Apicella et al., 1991b; Hollerman et al., 1998; Histed et al., 2009). Furthermore, there are striatal neurons coding which action is associated to reward and which action is not (Hollerman et al., 1998; Kawagoe et al., 1998; Figure 2C). This coding is independent of the stimuli indicating the action required to obtain reward (Kimchi and Laubach, 2009; Kimchi et al., 2009). Reward-predicting cues modulate the activity of caudate neurons (Kawagoe et al., 1998; Lauwereyns et al., 2002). After saccade execution up to 50% of neurons encode only the action, while around 20% of recorded neurons encode whether the action was rewarded or not and close to 40% of neurons are modulated by both movement and reward (Kobayashi et al., 2006; Lau and Glimcher, 2007). Together, these data suggest that striatal neurons response is modulated by action and reward. These responses are not limited to the moment of movement or reward receipt; rather they are present during cue and during reward expectation.

Most striatal neurons that respond during task performance show higher activity when a reward is expected compared to when no reward is expected (Hollerman et al., 1998). However, there are also neurons that are active preferentially after the monkey is instructed to not move to obtain reward (Hollerman et al., 1998). These data suggest that striatal neurons flexibly encode the type of action that will produce reward.

An action-value neuron tracks the value of one action, independent of the performed action. By tracking the value of different candidate actions and comparing their values an organism can decide to exploit the most valuable action or to explore the value of other actions. Samejima et al. (2005) were the first group to show that striatal neurons code action-value (Figure 2D). Neuronal activity tracked over time the value of performing one action regardless of the animal's choice. Later, Lau and Glimcher (2008) trained macaques to perform a matching task. In this task rewards are distributed probabilistically between two options and subjects match the frequency with which they choose one action with its reward probability (Herrnstein, 1961). This task opens the possibility of investigating the presence of action-value and chosen-value (i.e., value of the chosen action) neurons. Indeed, Lau found that caudate neurons code both action-value and chosen-value. These signals can inform decision making mechanisms.

In conclusion, the striatum contains neuronal activity related to movements, rewards and the conjunction of both movement and reward. These neuronal representations serve many functions like goal directed movements and decision making.

## Striatal activity during social behavior

### Social reward

Rewards are events or objects that elicit learning, elicit approach behavior and produce positive emotions (Schultz, 2004). Social rewards are just like any other rewards with the particularity that they occur in a social context. We propose a simple classification of social rewards using two axes: who acts and who receives reward. For example, observing others is a social reward (Anderson, 1998; Deaner et al., 2005) where the individual acts (observes) and receives reward (the social stimuli). Pro-social behavior refers to a preference to increase the welfare of others (Fehr and Camerer, 2007). Depending on individual social preferences these choices can be rewarding by themselves, e.g., in charitable giving (Harbaugh et al., 2007). Vicarious reward refers to the situation when observing someone else receive reward is rewarding in itself (Mobbs et al., 2009). Finally, in several social rewards the recipient is the individual and the actor is someone else. Examples of other's actions that are rewarding include praise and pleasant touch (Francis et al., 1999; Olausson et al., 2002; Rolls et al., 2008; Korn et al., 2012). Building a desired reputation is also considered a social reward; critically, reputation depends on other's perception of the individual, not on the individual's perception of herself (Izuma et al., 2008; Izuma, 2012). Receiving gifts or social actions that result in own reward can also be considered as other-generated social rewards. Social inclusion can be considered a social reward and facilitates learning (Eger et al., 2013). Although this classification might further our understanding of the neuronal underpinnings of social rewards, further experimentation might validate its use.

#### Observing others

Fuelling a brain entails a huge cost, and the ratio of brain size to body size is larger in primates than any other Order in the animal kingdom (Laughlin and Sejnowski, 2003; Dunbar and Shultz, 2007). The huge cost of fuelling a large brain begs the question what is the benefit of such large brains? Byrne and Whitten suggest that only a costly primate brain can deal with the complexity of primate social living, the so-called social brain hypothesis (Dunbar and Shultz, 2007). The primate brain has a great deal of specializations to acquire information about conspecifics. Neurons in the ventral visual pathway respond selectively to biological motion, gaze direction, body parts and faces (Perrett et al., 1984, 1985a,b; Gross, 1992; Oram and Perrett, 1996; Tsao et al., 2006). Social information arrives through all senses. For example, the superior temporal polysensory area contains neurons that selectively respond to conspecific calls (Perrodin et al., 2011) and local field potentials in the temporal lobe are modulated by face or call familiarity (Báez-Mendoza and Hoffman, 2009). The volume of gray matter correlates with the size of the individual's troop in mid superior temporal sulcus, inferotemporal cortex, rostral superior temporal sulcus, amygdala—all areas involved in perceiving individuals—and rostral PFC in macaques (Sallet et al., 2011). These findings suggest that the brain has specialized structures dealing with the acquisition and representation of information about conspecifics.

If the brain has specialized structures for the acquisition and representation of information about conspecifics, then acquiring this information must be valuable for the individual. In a clever paradigm Deaner and colleagues measured the value of acquiring access to observe pictures of conspecifics (Deaner et al., 2005). They pitted a constant amount of juice against a variable amount of juice plus the opportunity to observe the picture of a conspecific. The monkeys made their choices depending on the amount of juice offered along with the picture. If the monkey chose a smaller amount of juice plus the opportunity to watch an image, it strongly indicated that the monkey valued watching the image equivalent to the difference between offered juice volumes. For example, a monkey that likes watching a high-ranking monkey will choose watching the image and receiving 0.8 ml of juice vs. only receiving 1ml of juice. When the monkey chose with equal probability between the two alternatives then the difference in offered juice volume is the subjective value for observing the image, the so-called point of subjective equivalence. Researchers using this method can measure the subjective value of varying juice magnitudes (fluid value) and that of social images (image value). Another advantage of this method is that it facilitates the comparison of different goods (Glimcher, 2010), e.g., observing female perinea or a subordinate male face. Using this method Deaner and colleagues reported that male monkeys valued highly looking at dominant monkeys and the perinea of female monkeys compared to looking at subordinate monkeys or a non-salient visual stimulus (Deaner et al., 2005).

Neuronal activity during this task has been measured in different brain regions. LIP neuronal activity correlates with both image value and fluid value when the monkeys chose to look at the image (Klein et al., 2008). OFC neurons showed distinct coding of reward magnitude or image value, but not both (Watson and Platt, 2012). Thus, these results suggest that OFC neurons do not code reward on a single currency (e.g., in juice volume), rather as different variables, as shown before (O'Neill and Schultz, 2010). Intriguingly, these animals strongly preferred looking at pictures of subordinates, a finding at odds with previously reported strong preferences for dominant faces in the same paradigm (Deaner and Platt, 2003; Deaner et al., 2005; Shepherd et al., 2006; Klein et al., 2008); but this result suggests that the encoding of social reward reflects subjective preferences.

Neurons in the anterior striatum showed an interesting response pattern in the same paradigm (Klein and Platt, 2013). The large majority of reward responsive neurons were selective for reward type. These neurons also showed a regional pattern: those in the caudate were more strongly modulated by social reward, conversely, putamen neurons were more strongly modulated by liquid reward. This pattern can be alternatively explained by simple saccade direction coding because caudate neurons are tuned for saccade direction, particularly for contralateral saccades (Hikosaka et al., 1989a).

**Figure 3:**
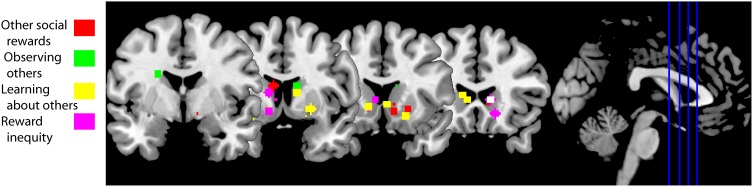
**fMRI studies of social behaviors in which the striatum is active**. Peak activation coordinates in the striatum of the fMRI studies cited in this review color-coded for each section as illustrated in the legend. Studies using a region of interest analysis strategy were not included in this image. These striatal responses are compatible with a general activation in response to social behaviors, including social rewards. A functional subdivisions according to types of social rewards need to await further experiments. Studies aggregated in “Other social rewards”: (Rilling et al., 2002; Moll et al., 2006; Izuma et al., 2008; Mobbs et al., 2009; Acevedo et al., 2012; Fareri et al., 2012; Korn et al., 2012). Studies clustered in “Observing others”: (Bartels and Zeki, 2000, 2004; Aron, 2005; Acevedo et al., 2012). Studies in “Learning about others”: (Delgado et al., 2005; King-Casas et al., 2005; Baumgartner et al., 2008; Burke et al., 2010; Phan et al., 2010; Xiang et al., 2012; Fouragnan et al., 2013). Studies in “Reward inequity”: (Moll et al., 2006; Fliessbach et al., 2007; Hsu et al., 2008; Tricomi et al., 2010).

Humans also value observing other humans; and among different targets we value highly observing our romantic partners and mothers (Bartels and Zeki, 2000, 2004; Aron, 2005; Acevedo et al., 2012). Observing pictures of a partner elicits higher blood oxygenated level-dependant (BOLD) activity in caudate/putamen and VTA along with cingulate and insular cortex compared to viewing pictures of friends matched for age, gender and length-of-friendship as their partners (Figure 3, green squares). This effect is present either when the relationship is recent (Aron, 2005) or when has been long established (Acevedo et al., 2012). These BOLD responses are a neural correlate of the value of observing a loved one.

In summary, acquiring social information, in particular looking at conspecifics, is valuable for the individual (Deaner et al., 2005). The primate temporal lobe contains regions whose function includes the processing of social information (Tsao et al., 2006; Perrodin et al., 2011). Both social information and value converge in the striatum, opening the possibility of social reward coding in this brain region—as shown by Klein and Platt (2013).

#### Other social rewards

A positive reputation is a social reward as it can elicit learning, approach behavior and positive emotions. This is particularly evident in indirect reciprocity: a donor who helps a recipient in public might receive in the future a donation from someone that has observed its “altruistic” behavior (Nowak, 2006). Obtaining a good reputation from others increases BOLD activity in the human striatum (Izuma et al., 2008; Korn et al., 2012) (Figure 3, red squares), but not in individuals diagnosed with autism (Izuma et al., 2011). This difference is likely due to insensitivity to social rewards in autistics (Dawson et al., 1998; Schultz, 2005).

Other social rewards that also increase BOLD activity in the striatum include charitable donations (Moll et al., 2006; Harbaugh et al., 2007) and observing someone else succeed (Mobbs et al., 2009). Vicarious reward is also modulated by the closeness of the recipient: there is higher striatal BOLD activity when sharing a monetary gain with close friends compared to sharing with strangers, and sharing with the latter is associated with higher activations compared to when the “recipient” is a computer (Fareri et al., 2012). This social vs. non-social effect has also been observed when cooperating with a human partner vs. cooperating with a computer (Rilling et al., 2002). The peak activations from studies cited in this section are illustrated with red squares in Figure 3. Taken together, these data suggest that social rewards are associated with BOLD activity in the striatum and can be modulated by the social context.

### Learning about social agents

Social life is rife with opportunities to learn about others. For example, we learn to trust or mistrust other people. The trust game is an economic game that measures how trust is built between two individuals. During the trust game the investor receives an initial endowment that she can choose to invest in a trustee, the trustee receives three times the investment and decides how much of the gains to return to the investor. When this game is played iteratively the investor learns to trust (or mistrust) the trustee and vice versa. Thus, both players develop a model of the other's reputation (King-Casas et al., 2005). To build a trust model investors use previous behavior to predict future behavior. If there is a deviation from what is predicted—a reward prediction error—then the model is updated. Activity in dorsal striatum mirrored prediction errors during the repayment phase (Figure 3, yellow squares; King-Casas et al., 2005). When an investor returned more than what a trustee expected the trustee reciprocated by increasing her investment. During the investment phase activity increased in middle cingulate cortex of the investor and also in ACC of the trustee. Activity in both areas correlated with activity in the trustee's caudate; most importantly the peak of these correlations shifted from the repayment epoch to the investment epoch (King-Casas et al., 2005). These results suggest that generating someone else's reputation engages a reinforcement learning algorithm that uses prediction errors and the latter are reflected in striatal BOLD activity.

Prior information about someone's trustworthiness sets the initial state of the trust model. This initial bias can be overruled by observing someone's willingness to reciprocate trust (Figure 3, yellow squares; Delgado et al., 2005; Phan et al., 2010; Fouragnan et al., 2013). Prior information diminishes the magnitude of the reward prediction error signal in the striatum during the repayment phase (Fouragnan et al., 2013). Following advice to solve a task (a type of prior information) generates an outcome-bonus in a version of the Iowa gambling task (Biele et al., 2011). These studies suggest that prior information not only sets the initial state of the trust model, but it has a long lasting effect on its computation.

Depth-of-thought refers to a person's inference about someone else's intention and to how many iterations of this inference they perform (Dixit and Skeath, 2004). Players in the trust game solve the game with different levels of depth-of-thought (Xiang et al., 2012). If the investor makes no inference about the trustee's intention to reciprocate, then a prediction error occurs when the trustee does not reciprocate trust. This prediction error is reflected in increased striatal activity (Figure 3, yellow squares; Xiang et al., 2012). If the investor infers that he plays this game against a trustee that infers what he will offer, then the prediction error occurs when the investor submits its investment to the trustee; again, the striatum reflects this prediction error (Xiang et al., 2012). Thus, the computation of prediction errors, during the trust game, depends on depth-of-thought.

Oxytocin, a neuropeptide, also modifies how we update the trust model. Intranasal administration of this neuropeptide increases the rate of trust decisions compared to placebo, even after repeated violations of trust (Kosfeld et al., 2005). Correspondingly, people that received oxytocin showed a smaller negative prediction error signal in the striatum after repeated violations of trust (Baumgartner et al., 2008). Although the distribution of oxytocin receptors in the human brain is unknown, one possible locus where oxytocin modifies trust is in the striatum (see section “Involvement of the Striatum in Pair-Bond Formation and Maintenance” below).

Social life is also rife with opportunities to learn from others. Observational learning is another social cognitive process that can be modeled with reinforcement learning. Burke and colleagues hypothesized that observational learning is composed of two prediction errors, an action observation prediction error and an outcome observation prediction error (Burke et al., 2010). In their task two individuals took turns to learn which one of two decks of cards provided a better outcome. In order to disentangle individual learning from imitation learning and observational learning the individuals performed the task in three conditions: other's actions and outcomes were private, only the other's outcome was visible and both the partner's action and outcome were observable. Burke and colleagues found a correlate for action observation prediction error in dorsolateral prefrontal cortex (DLPFC) and for outcome observation in ventromedial prefrontal cortex (VMPFC) and ventral striatum (Figure 3, yellow squares). Specifically, VMPFC activity correlated positively and ventral striatum correlated negatively with the outcome observation prediction error (Burke et al., 2010). Thus, they found neural correlates of observational learning in frontal cortex and ventral striatum.

In conclusion, the neuronal mechanism of learning to trust someone else or from someone else is based on a reinforcement learning algorithm. This algorithm makes predictions about other's behavior and prediction errors help to update the model. The type of predictions depends on depth-of-thought and prior information modifies the rate to which the model is updated. These learning signals are reflected in changes in BOLD activity in the striatum.

### Inequity and fairness considerations

Inequity arises from an asymmetric distribution of resources between two or more conspecifics. Classic economics assumes that agents always intend to maximize their own benefit regardless of other's wellbeing (Von Neumann and Morgenstern, 1947). However, the difference in resource distribution can have a negative impact on the utility and subjective value of an object (Loewenstein et al., 1989; Fehr and Schmidt, 1999). The disutility from an unequal outcome depends on who obtains more resources. When the agent receives more than the conspecific, we speak of advantageous inequity. Conversely, when the agent receives less than the conspecific we speak of disadvantageous inequity.

Interestingly, humans choose to lower their own payoff so that inequity is smaller, a so-called pro-social behavior. For example, when people donate money to charity they diminish their wealth so that others can be better off (Harbaugh et al., 2007). Disadvantageous inequity, having less than others, can have a negative effect in behavior. For example, progressive taxation is designed to reduce income inequality by implementing higher taxes on higher earners (Wilkinson and Pickett, 2010). An influential hypothesis of how people react to inequity (Fehr and Schmidt, 1999) posits that unequal payoffs are aversive, therefore agents try to minimize them. This theory has its roots on the idea that one can estimate social utility functions that specify level of satisfaction as a function of outcome to self and other (Loewenstein et al., 1989). Other example theories where social utility functions help to explain human preferences that deviate from pure maximization include “Equity, Reciprocity, and Competition” by Bolton and Ockenfels (Bolton and Ockenfels, 2000) and “Fairness” by Rabin (Rabin, 1993).

One experimental task commonly used to measure advantageous inequity aversion is the dictator game (Forsythe et al., 1994). In this task the person playing as dictator receives an initial financial endowment and decides to give an amount of the endowment to a receiver. The neoclassical assumption of rational behavior predicts that dictators will not give away anything of their payoff; however, dictators usually give away between 5 and 25% of their initial endowment (Forsythe et al., 1994). It is assumed that the proportion of money given to the receiver is a measure of the disutility for the dictator of having more than the other (Gibbons, 1992; Camerer et al., 2004). To measure disadvantageous inequity aversion scientists use the ultimatum game (Güth et al., 1982). In this game the proposer receives an endowment and proposes a split to the responder, just as in the dictator game. The responder then either rejects the split, thereby forgoing all monies, or accepts it. Neoclassical economic models predict that the responder will accept any split that results in him having more than nothing. However, responders tend to only accept splits where they obtain more than 30% of the initial endowment (Güth et al., 1982). The responder's minimum acceptable offer is the percentage of the initial endowment that he is willing to accept 50% of the time (Camerer et al., 2004). This last parameter is directly proportional to the degree of disadvantageous inequity aversion.

When subjects play the dictator game as dictators the ventral striatum is active when deciding to donate money to a charity (Moll et al., 2006; Harbaugh et al., 2007) and when enacting the decision on how to distribute a good between two charitable possibilities (Hsu et al., 2008). The relative wealth of the donor and the receiver also matter to how the brain responds to these decisions. After one of two volunteers is made better-off than the other volunteer, the worse-off volunteers ranked receiving money much more appealing than their better-off counterparts (Tricomi et al., 2010). Accordingly, ventral striatum and VMPFC show higher activity during transfers to self than to the other. Better-off volunteers found more appealing that the other received money than themselves. Ventral striatum and VMPFC reflected this preference: both brain regions showed higher activity during transfers to other than to self (Tricomi et al., 2010). In a related experiment, Fliessbach and colleagues paid in different ratios to pairs of volunteers for correctly completing a simple task while they were in an MRI scanner (Fliessbach et al., 2007). Ventral striatum activity was positively correlated with the ratio of the payoff regardless of the actual personal monetary payoff. Furthermore, striatal activity was lowest during own errors and highest during other's errors. Such a social contrast has been confirmed, e.g. activity in ventral striatum is higher after winning a lottery in public vs. winning the same amount in private (Bault et al., 2011). The peak activations from the fMRI studies cited in this section are illustrated in Figure 3 with pink squares. Thus, these data suggest that the striatum reflects the difference between own and other's rewards.

### Agency coding in striatal neurons

Reciprocal social interactions provide the opportunity to increase fitness through repeated exchanges with a particular individual, although one of its by-products is reward inequality. For this interaction to be successful several mental processes need to take place (Axelrod and Hamilton, 1981): both participants need to identify their partner, assign agency for the current outcome, decide how to act depending on the series of events and keep a tally of the recent exchanges. Without partner identification reciprocity is virtually impossible (unless all interactions take place with a uniform population) (Dawkins, 2006). Without a memory trace of the outcomes of the recent exchanges, participants might see themselves locked onto a “one-way street” reciprocal exchange. Agency assignment allows the individual to assign credit (or blame) for a shared outcome (Wolpert et al., 2003; Tomlin et al., 2006). With precise agency assignment in the memory of recent exchanges individuals can avoid free riders (Dawkins, 2006). Therefore, agency assignment is a trait that might have been favored by evolution in social animals.

**Figure 4:**
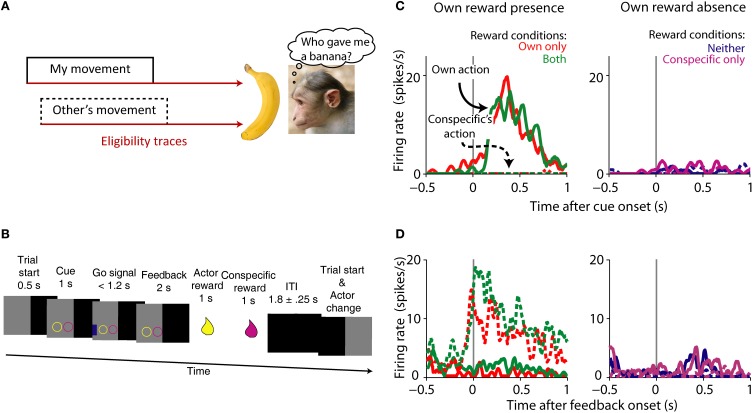
**Agency credit assignment cartoon and striatal neurons coding social action and own reward. (A)** Once the monkey receives a banana it needs to know which action produced reward to assign credit. The action can be its own (solid lines) or someone else's (dashed lines). Many actions take place before reward is delivered, therefore looking at a memory of each action or eligibility trace (brown arrows) can solve the agency credit assignment problem. **(B)** Task sequence for the actor: shape of conditioned cue predicted absence or presence of reward for each animal. Appearance of a subsequent blue go signal was followed by key release, stimulus touch and reward for actor, and later for conspecific. After the ITI the monkeys switched roles as actor and passive. **(C)** Single striatal neuron coding own action and own reward. Note the higher neuronal activity during own action and own reward compared to own reward absence and conspecific's actions. **(D)** Single striatal neuron coding social action and own reward. This neuron is active during conspecific's actions that will result in own reward, a complement to the neuron shown in **(A)**. Monkey picture by smerikal (Flickr), reproduced with permission. Panels **(B–D)** based on Báez-Mendoza et al. (2013), reproduced with permission.

Another way to frame the problem of agency assignment is to think of it as the “social” extension of the credit-assignment problem (Figure 4A). Let us revise what the credit-assignment problem is. In order for an action to be reinforced, it needs to be selected from various actions made between the operant and the reinforce. The organism needs to assign credit to the operant, and not assign (or subtract) credit to other non-contingent actions (Sutton and Barto, 1998). This is done by changing the weights of different eligibility traces, or memories of past actions (Sutton and Barto, 1998). The agency credit assignment problem applies when more than one actor can generate a reward (Tomlin et al., 2006). Thus, the agency credit assignment problem can be cast by paraphrasing Sutton and Barto (1998): how do you distribute credit for success among the many *actors* that may have been involved in producing it?

The striatum is well-suited for integrating social action (an action made in a social context) and reward given its anatomical connections and known role in action and reward coding. We recorded striatal neuron's activity while an animal performed a reward giving task with a conspecific in order to investigate the interaction of social action and reward (Báez-Mendoza et al., 2013). The reward giving task is an extension of the paradigm described by Hollerman et al. (1998) to encompass several social dimensions. In the original paradigm the activity of striatal neurons was tested for relationships to movement vs. no-movement and reward vs. no-reward. In our task we tested if striatal neuron activity was related to own vs. conspecific's movement and own and/or conspecific's reward. During the experiment two monkeys sat opposite each other across a table with a touchscreen. Both animals took turns to complete the following task: the actor held a resting key with its right arm, the computer presented two simultaneous cues predicting reward (circle) or no reward (square) separately for each animal (Figure 4B), followed by a blue go signal eliciting the actor's arm movement for touching it (Figure 4B). After a brief delay, the computer delivered reward to the actor and then to the conspecific. We were able to probe the neuronal correlates of agency and reward coding by varying reward presence and absence for both players and who performed the task. This simple test allowed us to test the neuronal mechanisms of a complex cognitive process.

Our first concern was whether the monkeys were sensitive to the social nature of the task. Reaction times and eye fixation analysis suggested that the monkeys were sensitive to reward received by themselves and their conspecific. Importantly, the animals were less likely to move whenever it was the conspecific's turn, suggesting that they had an understanding of the turn-taking structure of the task. This is particularly relevant for agency credit assignment because during “own turns” the animal should have assigned credit to itself for own reward and during “conspecific's turns” to the conspecific.

Own reward modulated the activity of striatal neurons, as previously observed (Hikosaka et al., 1989b; Apicella et al., 1991a); but few striatal neurons responded to conspecific's reward. Interestingly, a sub-population of neurons differentiated between social actors, with some neurons firing more strongly during one of the actor's turn. Given these types of neuronal modulations, we then looked at the neurons' sensitivity to whose turn it was. A large number of own reward coding neurons reflected the social actor: some neurons responded to own reward only when the recorded animal acted (Figure 4C) whereas a different sub-population responded to own reward when the conspecific acted (Figure 4D). We tested a series of alternative hypothesis for these data including: eye position, response inhibition, temporal discounting and reward cost, none of which were a satisfactory explanation of the data.

We also found a collection of neurons that reflected whose trial it was. These neurons fired more strongly during own trials than conspecific's trials, or vice versa: conspecific > own trials. These neurons reflected social action as they differentiated between actors. To test whether these neurons truly reflected a “social” component of the task we measured their activity while the animal performed the task with the conspecific or a non-social juice recipient (an empty bucket). If a neuron is modulated by the social component of the task, then it should stop differentiating between actors during the “bucket test.” This test for social-specific coding indicated that close to 50% of social actor coding-neurons were indeed modulated by the social environment. This is, to our knowledge, the first direct test of a neuronal correlate of social behavior in single neurons.

These experiments showed that there are multiple signals in the striatum relevant for social interactions. The data suggests an extension of the known role of the striatum in movement and reward processing into the social domain. Several questions arise from these findings.

How are these signals formed? One possible mechanism is as follows: Striatal neurons receive biological motion information either directly from area STP (Oram and Perrett, 1996) or indirectly via parietal lobe (Cavada and Goldman-Rakic, 1991) while simultaneously receiving reward-related information from dopaminergic neurons and other reward-related areas (Haber and Knutson, 2010, see also Figure 1). Converging inputs and local interactions (Chuhma et al., 2011) are also well-suited to combine information about other's actions and own reward. Future experiments will test and measure the formation of agency and reward conjoint coding in the population of striatal neurons.

Another issue is: how are these signals used? We hypothesize that this neuronal signal may help assign, and maintain, credit to a social agent when receiving reward in a social context. Solving this problem is necessary for successful interactions. It is possible the striatum provides a signal to distribute credit for reward among the many actors that may have been involved in producing it. One key experiment would test the individual-specificity of this signal: is the signal specific for one individual or it only discriminates between own action and “other's” actions? Such a fine grained signal would aid in discriminating who is a better partner and who is not.

### Social contact and striatal function

The striatum is involved in other social behaviors besides social action, social reward and reward inequity. Social isolation and social defeat compromise the normal function of the striatum. These effects highlight the interplay between normal social contact and striatal function. Social isolation has long-lasting effects in behavior, neuronal anatomy and neurochemistry. For example, social deprivation in the first year of life of macaques is related to abnormal social behaviors including fearfulness, withdrawal, lack of play, apathy, indifference to external stimuli, deficiencies in communication and aggression (Martin et al., 1991). Macaques reared in social deprivation show decreased numbers of caudate/putamen neurons reactive to substance P, tyrosine hydroxylase (TH), leucine-enkephaline, and calbindin; in contrast, the number of somatostatin interneurons did not differ to normally-reared conspecifics. TH staining was reduced in SNc but neuron numbers were stable. Other subcortical regions were unaffected, including the NAcc, amygdala and BNST (Martin et al., 1991). Further characterization of the behavioral, anatomical and neurochemical effects of social isolation have been carried out in rodents.

Social isolation leaves consistent behavioral effects on rodents. These include hyper-reactivity to novel environments, a reduction in the pre-pulse inhibition of the acoustic startle, and an increase in aggressive behavior (reviewed by Fone and Porkess, 2008). Also, studies of the neuroanatomy of isolates' brains describe changes in cortical and subcortical neuronal circuits. For example, after social isolation rats showed decreased dendritic spine density in prefrontal cortex and hippocampus compared to socially-housed littermates (Silva-Gomez et al., 2003). There are several reports on differences in neurotransmitter systems, for a systematic review see (Fone and Porkess, 2008). Of particular relevance to this review, the dopaminergic system of socially isolated rats is different to that of socially-housed animals.

Although socially isolated rats show normal basal levels of extracellular dopamine (DA) in the ventral striatum, systemic administration of d-amphetamine produces a significant increase in DA release compared to socially-reared rats (Wilkinson et al., 1994; Hall et al., 1999). Furthermore, isolation-reared rats show an increase in DA turnover and in hyper-locomotion induced by d-amphetamine (Hall et al., 1998). Injections of cocaine increase DA efflux in ventral striatum, an effect potentiated by isolation rearing (Howes et al., 2000). Intriguingly, isolates acquire faster operant responding to obtain low doses of cocaine but their acquisition is slower for higher doses compared to socially-housed rats (Howes et al., 2000). Deficits in pre-pulse inhibition of the acoustic startle in socially-isolated rats are reversed by administration of the D2 receptor antagonist raclopride (Geyer et al., 1993). DA depletion in ventral striatum after administration of 6-hydroxydopamine also facilitates pre-pulse inhibition in socially-isolated rats (Powell et al., 2003). Interestingly, basal levels of extracellular DA in ventral striatum do not differ between socially-isolated and socially-reared rats (Wilkinson et al., 1994; Hall et al., 1999; Howes et al., 2000). These results suggest that basal mesolimbic DA is unaffected by social isolation, rather the ventral striatum is “hypersensitive” to events that naturally trigger DA release.

One candidate mechanism for the hypersensitive ventral striatum of socially-isolated rats is a difference in receptor levels. Yet some groups report no changes in D1 or D2 receptor density or affinity in striatum (Bardo and Hammer, 1991; Del Arco et al., 2004); while others report an increase in D2 binding (Djouma et al., 2006). Changes in housing condition, however, modify the levels of D2 receptors in the monkey striatum (Morgan et al., 2002). Specifically, after monkeys were socially housed, dominant monkeys had higher levels of D2 receptors in striatum compared to when they were housed individually and to subordinates. Interestingly, subordinates consumed more and worked more for intravenous injections of cocaine than dominant monkeys (Morgan et al., 2002). This finding is further supported by a negative correlation between the baseline levels of D2 receptors and the rate of cocaine self-administration and a decrease in D2 receptor levels with chronic cocaine use (Nader et al., 2006). Thus, these results suggest that D2 receptor density can be modified by changes in the social environment.

Changes in social hierarchy result in winners and losers: lower ranking individuals were usually defeated by their conspecifics and lost their rank. After losing one or more encounters with a conspecific, mesostriatal transmission is modified in the defeated individual. Tidey and Miczek (1996) reported that rats that were defeated by a conspecific, showed higher concentrations of extracellular DA in ventral striatum and prefrontal cortex during a social encounter with a dominant rat compared to baseline. If rats remained isolated after being defeated, the number of striatal dopamine transporter (DAT) binding sites was reduced, while there were no changes in DAT in animals that returned to the familiar group (Isovich et al., 2001). A potential role of levels of DAT in regulation of social behavior is suggested by a report of DAT knockout mice which exhibited increased rates of reactivity and aggression following mild social contact (Rodriguiz et al., 2004). Mice who experienced chronic social defeat avoid making contact with conspecifics and show increased levels of brain derived neurotrophic factor (BDNF) in the NAcc up to 4 weeks after the last defeat (Berton et al., 2006). BDNF potentiates DA release in the NAcc by acting in pre- and post-synaptic sites (Russo and Nestler, 2013). The major source of BDNF in NAcc is dopaminergic neurons in VTA. BDNF deletion in these cells of chronically-defeated mice results in an increase in social contact, suggesting that BDNF plays a key role in the maintenance of the social defeat phenotype (Berton et al., 2006). These selected studies highlight that mesolimbic dopaminergic transmission is modified following acute or chronic social defeats.

In conclusion there are behavioral, anatomical and neurochemical consequences of social isolation. There is a marked reduction in the number of striatal interneurons, but basal levels of extracellular DA remain unchanged. There is no consensus whether there are changes in DA receptor levels in the striatum, but other signaling systems (BDNF) and molecular mechanisms (changes in DAT) are involved. This snapshot of studies on the relationship between social housing conditions, behavior and basal ganglia function suggest that this is not a simple relationship. Notwithstanding, it can be concluded that social isolation and social defeat result in changes in neurotransmission to the mesolimbic circuit.

### Involvement of the striatum in pair-bond formation and maintenance

Sex is a primary reward and it is the basis of pair-bond formation in voles. The striatum is part of the neuronal circuitry underlying a remarkable pair-bond formation in which both partners remain monogamous. It is important to note that the role of the striatum extends beyond that of movement and reward. Studies on vole pair formation provide an interesting example of the interaction between social behavior and striatal function.

There are two similar species in the same genus: one of which is monogamous and the other promiscuous. Prairie voles (*Microtus ochrogaster*) form life-long bonds with their first mate, remain monogamous and live in burrows with extended families; meadow voles (*Microtus pennsylvanicus*), in contrast, are a promiscuous species often living in solitary burrows (Insel, 2010). This natural dissociation in pair formation provides the opportunity to tap into the neurobiology of social behavior.

The interplay of oxytocin, arginine-vasopressin and DA play a pivotal role in pair formation in voles. Administration of haloperidol—an unselective DA inverse agonist—in male prairie voles' NAcc prevents partner preference, whilst stimulating D2-like receptors in caudate-putamen induces partner preference in the absence of mating (Aragona et al., 2003, 2006). Conversely, DA D1-like receptor activation prevents pair-bond formation (Aragona et al., 2006). This mechanism is similar in females, since D2-like receptor stimulation induces partner preference whereas administration of a D1-like agonist had no effect (Wang et al., 1999). Vasopressin V1a receptor gene transfer into the ventral pallidum of polygamous meadow voles is sufficient to induce pair-bond-like behavior after mating (Lim et al., 2004b). Similarly, overexpression of oxytocin receptor in NAcc facilitated partner preference in female prairie voles but has no effect in parental care, nor any effect on female meadow voles (Ross et al., 2009). Prairie voles have a high density of oxytocin-receptors in the NAcc and of vasopressin V1a receptors in the ventral pallidum compared to meadow voles (Insel and Shapiro, 1992; Hammock and Young, 2006). Interestingly, oxytocin-receptors are bound by oxytocin, and with lower affinity, vasopressin (Gimpl and Fahrenholz, 2001). Interestingly, there are no differences in the distribution of D1-like and D2-like receptors in the striatum between these two species (Lim et al., 2004a). Thus, these results suggest that the differential distribution of oxytocin and vasopressin receptors is responsible for pair-bond formation. In conclusion, pair-bond formation is modulated by the interaction of oxytocin, vasopressin and DA in NAcc neurons as well as the distribution of oxytocin and vasopressin V1a receptors.

The role of oxytocin and vasopressin in social recognition is supported further by the absence of habituation to conspecifics in oxytocin and V1a-R knockout mice (Ferguson et al., 2000; Bielsky et al., 2004). Oxytocin knockout mice “recover” social habituation after infusion of oxytocin agonists in central amygdala (Ferguson et al., 2001). Similarly, local infusion of V1a-R antagonists in lateral septum of rats inhibits habituation to conspecifics (Everts and Koolhaas, 1999). Thus, both oxytocin and vasopressin regulate social recognition.

The endogenous opioid system is another neuronal mechanism that may play a role in pair-bond formation. Mu-opioid receptor (MOR) activation modulates partner preference in female prairie voles (Burkett et al., 2011). MOR density is striatal region specific, thus this effect is probably mediated by specific striatal regions (Resendez et al., 2013). MORs within the dorsal striatum mediate partner preference formation via impairment of mating, whereas receptors in NAcc appear to mediate pair bond formation through the positive hedonics associated with mating (Resendez et al., 2013). Interestingly, monogamous voles show higher MOR density in forebrain including the caudate-putamen and NAcc than the closely-related polygamous voles (Inoue et al., 2013), but see (Insel and Shapiro, 1992). Thus, interspecies differences in opiate receptor density and pharmacological effects suggest a role of opiates in social attachment.

A relevant question is how and where these neurotransmitter systems interact. Rat NAcc core neurons expressing D1-like receptors co-express prodynorphin, conversely D2-like expressing cells co-express proenkephalin (Curran and Watson, 1995). An electron microscope investigation indicates that about half of neurons in the rat dorsolateral striatum co-express D2 and MORs (Ambrose et al., 2004). These anatomical studies support the possibility that oxytocin, vasopressin and D2-like receptors are present in single striatal cells, yet their interactions remain to be further investigated.

Little is known about pair-bond formation in primates. However, marmosets, a monogamous new-world monkey, show oxytocin receptor labeling in NAcc among other subcortical structures (Schorscher-Petcu et al., 2009), whereas rhesus macaques, a polygamous old-world monkey, only show labeling for this receptor in hypothalamus and the nucleus basalis of Meynert (Freeman et al., 2012). Titi monkeys are a monogamous species that exhibit small, but significant, changes in glucose intake in the NAcc and ventral pallidum 48 hr. after mating (Bales et al., 2007).

Whereas we have learned about pair-bond formation, the neuronal mechanisms of pair-bond maintenance are just starting to be investigated. For example, monogamous male voles show a significant increase in D1-like receptors in NAcc after pair-bond formation, and D1-like receptor antagonists diminish aggressive behavior toward female strangers—a behavioral marker of pair bond formation (Aragona et al., 2006). This is probably the most exciting open question in pair-bond formation, what are the neuronal mechanisms of pair-bond maintenance?

The striatum might also play a role in mother's recognition of offspring. The pregnancy hormones progesterone and oestrogen prime the brain for the synthesis of oxytocin and its receptor (Keverne and Curley, 2004). Olfaction is the prime sense for maternal offspring recognition in mammals. Oxytocin receptors expression increases in central olfactory projections and NAcc during pregnancy (Keverne and Curley, 2004).

Overall, these studies suggest a mechanism for pair-bonding formation in voles. The hypothetical mechanism is centered in the striatum's capability to facilitate the association between olfactory social cues and reward. A potential mate's pheromones reach the vomeronasal organ (VNO), which in turns transmits the individual's information to the extended amygdala and the central amygdala further transmits this information to striatum. VNO lesions in female voles disrupt pair formation (Curtis et al., 2001), a finding that supports this hypothetical mechanism. However, other brain areas may also play a role in pair-bond formation. For example there are marked differences in the distribution of dopamine, oxytocin and vasopressin receptors in the medial prefrontal cortex of monogamous and promiscuous voles (Smeltzer et al., 2006). As noted by Wang and Young (Lim et al., 2004b; Young and Wang, 2004), the cellular mechanism might be the co-activation of D2-expressing accumbal neurons by vasopressin and/or oxytocin. Oxytocin is released by the hypothalamus, odor information transmitted from the central amygdala and DA is released by dopaminergic neurons in VTA. Striatal neurons are well-suited for detecting the conjunction of sensorimotor information and reward. In pair-bond formation the role of the striatum, particularly the NAcc is to facilitate the association of social cues and reward to guarantee reproductive success.

## Conclusions

Based on the studies reviewed here, we conclude that the striatum plays a role in computations that take place during social behavior. These computations revolve around social actions and social rewards. fMRI and neurophysiology studies show that neural activity in the striatum is modulated by social rewards and by learning in a social context (Figure 3). By learning in this context we refer to: learning about other's preferences, a new mate, about other's actions that lead to own reward, or updating our predictions about other's preferences. We have shown that neuronal activity in the striatum is also modulated by social actions and, critically, by the conjunction of social action and own reward (Figure 4). The computations performed by the striatum are critical for successful social interactions. A breakdown in social interactions leads to compromised striatal function, which highlights the interplay between this neuronal circuit and social behavior.

Overall, these observations suggest that the striatum does not appear to have a particular “social” specialization; rather its neurons are capable of flexibly incorporating social information into their computations. Therefore, it is justified to speak of the striatum as containing a general purpose neuronal mechanism to associate actions or events with reward. Importantly, it can also associate—or reflect—other's actions to the rewards they lead to. Rewards are also coded in the activity of striatal neurons, and as social rewards are a sub-class of rewards, they are processed in the striatum. Importantly, a functional subdivision based on different types of social behaviors need to await further experimentation. In conclusion, the striatum plays a role in the computation of social behavior.

### Conflict of interest statement

The authors declare that the research was conducted in the absence of any commercial or financial relationships that could be construed as a potential conflict of interest.

## References

[B1] Acevedo B. P. Aron A. Fisher H. E. Brown L. L. (2012). Neural correlates of long-term intense romantic love. Soc. Cogn. Affect. Neurosci. 7, 145–159 10.1093/scan/nsq092 21208991 PMC3277362

[B2] Ambrose L. M. Unterwald E. M. Van Bockstaele E. J. (2004). Ultrastructural evidence for co-localization of dopamine D2 and μ-opioid receptors in the rat dorsolateral striatum. Anat. Rec. A Discov. Mol. Cell. Evol. Biol. 279A, 583–591 10.1002/ar.a.20054 15224400

[B3] Anderson J. R. (1998). Social stimuli and social rewards in primate learning and cognition. Behav. Process. 42, 159–175 10.1016/S0376-6357(97)00074-0 24897460

[B4] Apicella P. Ljungberg T. Scarnati E. Schultz W. (1991a). Responses to reward in monkey dorsal and ventral striatum. Exp. Brain Res. 85, 491–500 10.1007/BF00231732 1915708

[B5] Apicella P. Scarnati E. Schultz W. (1991b). Tonically discharging neurons of monkey striatum respond to preparatory and rewarding stimuli. Exp. Brain Res. 84, 672–675 10.1007/BF00230981 1864338

[B6] Apicella P. Scarnati E. Ljungberg T. Schultz W. (1992). Neuronal activity in monkey striatum related to the expectation of predictable environmental events. J. Neurophysiol. 68, 945–960 1432059 10.1152/jn.1992.68.3.945

[B7] Aragona B. J. Liu Y. Curtis T. Stephan F. K. Wang Z. X. (2003). A critical role for nucleus accumbens dopamine in partner-preference formation in male prairie voles. J. Neurosci. 23, 3483–3490 12716957 10.1523/JNEUROSCI.23-08-03483.2003 PMC6742315

[B8] Aragona B. J. Liu Y. Yu Y. J. Curtis J. T. Detwiler J. M. Insel T. R. (2006). Nucleus accumbens dopamine differentially mediates the formation and maintenance of monogamous pair bonds. Nat. Neurosci. 9, 133–139 10.1038/nn1613 16327783

[B9] Aron A. (2005). Reward, motivation, and emotion systems associated with early-stage intense romantic love. J. Neurophysiol. 94, 327–337 10.1152/jn.00838.2004 15928068

[B10] Axelrod R. Hamilton W. D. (1981). The evolution of cooperation. Science 211, 1390–1396 10.1126/science.7466396 7466396

[B11] Báez-Mendoza R. Harris C. J. Schultz W. (2013). Activity of striatal neurons reflects social action and own reward. Proc. Natl. Acad. Sci. U.S.A. 110, 16634–16639 10.1073/pnas.1211342110 24062436 PMC3799314

[B12] Báez-Mendoza R. Hoffman K. L. (2009). Object ontology in temporal lobe ensembles, in Cortical Mechanisms of Vision, 1st Edn., eds Jenkin M. Harris L. (Cambridge: Cambridge University Press), 237–253

[B13] Bales K. L. Mason W. A. Catana C. Cherry S. R. Mendoza S. P. (2007). Neural correlates of pair-bonding in a monogamous primate. Brain Res. 1184, 245–253 10.1016/j.brainres.2007.09.087 17976540 PMC2387250

[B14] Bardo M. T. Hammer R. P. (1991). Autoradiographic localization of dopamine D1 and D2 receptors in rat nucleus accumbens. Resistance to differential rearing conditions. Neuroscience 45, 281–290 10.1016/0306-4522(91)90226-E 1762680

[B15] Bartels A. Zeki S. (2000). The neural basis of romantic love. Neuroreport 11, 3829–3834 10.1097/00001756-200011270-00046 11117499

[B16] Bartels A. Zeki S. (2004). The neural correlates of maternal and romantic love. Neuroimage 21, 1155–1166 10.1016/j.neuroimage.2003.11.003 15006682

[B17] Bault N. Joffily M. Rustichini A. Coricelli G. (2011). Medial prefrontal cortex and striatum mediate the influence of social comparison on the decision process. Proc. Natl. Acad. Sci. U.S.A. 108, 16044–16049 10.1073/pnas.1100892108 21896760 PMC3179055

[B18] Baumgartner T. Heinrichs M. Vonlanthen A. Fischbacher U. Fehr E. (2008). Oxytocin shapes the neural circuitry of trust and trust adaptation in humans. Neuron 58, 639–650 10.1016/j.neuron.2008.04.009 18498743

[B19] Beckstead R. M. Domesick V. B. Nauta W. J. H. (1979). Efferent connections of the substantia nigra and ventral tegmental area in the rat. Brain Res. 175, 191–217 10.1016/0006-8993(79)91001-1 314832

[B20] Berton O. Mcclung C. A. Dileone R. J. Krishnan V. Renthal W. Russo S. J. (2006). Essential role of BDNF in the mesolimbic dopamine pathway in social defeat stress. Science 311, 864–868 10.1126/science.1120972 16469931

[B21] Biele G. Rieskamp J. Krugel L. K. Heekeren H. R. (2011). The neural basis of following advice. PLoS Biol. 9:e1001089 10.1371/journal.pbio.1001089 21713027 PMC3119653

[B22] Bielsky I. F. Hu S. B. Szegda K. L. Westphal H. Young L. J. (2004). Profound impairment in social recognition and reduction in anxiety-like behavior in vasopressin V1a receptor knockout mice. Neuropsychopharmacology 29, 483–493 10.1038/sj.npp.1300360 14647484

[B23] Bolton G. E. Ockenfels A. (2000). ERC: a theory of equity, reciprocity, and competition. Am. Econ. Rev. 90, 166–193 10.1257/aer.90.1.166

[B24] Burke C. J. Tobler P. N. Baddeley M. Schultz W. (2010). Neural mechanisms of observational learning. Proc. Natl. Acad. Sci. U.S.A. 107, 14431–14436 10.1073/pnas.1003111107 20660717 PMC2922583

[B25] Burkett J. P. Spiegel L. L. Inoue K. Murphy A. Z. Young L. J. (2011). Activation of mu-opioid receptors in the dorsal striatum is necessary for adult social attachment in monogamous prairie voles. Neuropsychopharmacology 36, 2200–2210 10.1038/npp.2011.117 21734650 PMC3176565

[B26] Calzavara R. Mailly P. Haber S. N. (2007). Relationship between the corticostriatal terminals from areas 9 and 46, and those from area 8A, dorsal and rostral premotor cortex and area 24c: an anatomical substrate for cognition to action. Eur. J. Neurosci. 26, 2005–2024 10.1111/j.1460-9568.2007.05825.x 17892479 PMC2121143

[B27] Camerer C. Loewenstein G. Rabin M. (2004). Advances in Behavioral Economics. Princeton, NJ: Russell Sage Foundation; Princeton University Press

[B28] Cavada C. Goldman-Rakic P. S. (1991). Topographic segregation of corticostriatal projections from posterior parietal subdivisions in the macaque monkey. Neuroscience 42, 683–696 10.1016/0306-4522(91)90037-O 1720224

[B29] Chuhma N. Tanaka K. F. Hen R. Rayport S. (2011). Functional connectome of the striatal medium spiny neuron. J. Neurosci. 31, 1183–1192 10.1523/JNEUROSCI.3833-10.2011 21273403 PMC3074638

[B30] Curran E. J. Watson S. J. (1995). Dopamine receptor mRNA expression patterns by opioid peptide cells in the nucleus accumbens of the rat: a double *in situ* hybridization study. J. Comp. Neurol. 361, 57–76 10.1002/cne.903610106 8550882

[B31] Curtis J. T. Liu Y. Wang Z. (2001). Lesions of the vomeronasal organ disrupt mating-induced pair bonding in female prairie voles (*Microtus ochrogaster*). Brain Res. 901, 167–174 10.1016/S0006-8993(01)02343-5 11368964

[B32] Dawkins R. (2006). The Selfish Gene: – with a New Introduction by the Author. Oxford: University Press

[B33] Dawson G. Meltzoff A. N. Osterling J. Rinaldi J. Brown E. (1998). Children with autism fail to orient to naturally occurring social stimuli. J. Autism Dev. Disord. 28, 479–485 10.1023/A:1026043926488 9932234

[B34] Deaner R. O. Khera A. V. Platt M. L. (2005). Monkeys pay per view: adaptive valuation of social images by rhesus macaques. Curr. Biol. 15, 543–548 10.1016/j.cub.2005.01.044 15797023

[B35] Deaner R. O. Platt M. L. (2003). Reflexive social attention in monkeys and humans. Curr. Biol. 13, 1609–1613 10.1016/j.cub.2003.08.025 13678591

[B36] Del Arco A. Zhu S. Terasmaa A. Mohammed A. H. Fuxe K. (2004). Hyperactivity to novelty induced by social isolation is not correlated with changes in D2 receptor function and binding in striatum. Psychopharmacology (Berl.) 171, 148–155 10.1007/s00213-003-1578-8 13680076

[B37] Delgado M. R. Frank R. H. Phelps E. A. (2005). Perceptions of moral character modulate the neural systems of reward during the trust game. Nat. Neurosci. 8, 1611–1618 10.1038/nn1575 16222226

[B38] Dixit A. K. Skeath S. (2004). Games of Strategy. New York, NY: W.W. Norton

[B39] Djouma E. Card K. Lodge D. J. Lawrence A. J. (2006). The CRF1 receptor antagonist, antalarmin, reverses isolation-induced up-regulation of dopamine D-2 receptors in the amygdala and nucleus accumbens of Fawn-Hooded rats. Eur. J. Neurosci. 23, 3319–3327 10.1111/j.1460-9568.2006.04864.x 16820021

[B40] Dunbar R. I. M. Shultz S. (2007). Evolution in the social brain. Science 317, 1344–1347 10.1126/science.1145463 17823343

[B41] Eger E. Moretti L. Dehaene S. Sirigu A. (2013). Decoding the representation of learned social roles in the human brain. Cortex 49, 2484–2493 10.1016/j.cortex.2013.02.008 23528247

[B42] Everts H. G. J. Koolhaas J. M. (1999). Differential modulation of lateral septal vasopressin receptor blockade in spatial learning, social recognition, and anxiety-related behaviors in rats. Behav. Brain Res. 99, 7–16 10.1016/S0166-4328(98)00004-7 10512567

[B43] Fareri D. S. Niznikiewicz M. A. Lee V. K. Delgado M. R. (2012). Social network modulation of reward-related signals. J. Neurosci. 32, 9045–9052 10.1523/JNEUROSCI.0610-12.2012 22745503 PMC3412567

[B44] Fehr E. Camerer C. F. (2007). Social neuroeconomics: the neural circuitry of social preferences. Trends Cogn. Sci. 11, 419–427 10.1016/j.tics.2007.09.002 17913566

[B45] Fehr E. Schmidt K. M. (1999). A theory of fairness, competition, and cooperation. Q. J. Econ. 114, 817–868 10.1162/003355399556151

[B46] Ferguson J. N. Aldag J. M. Insel T. R. Young L. J. (2001). Oxytocin in the medial amygdala is essential for social recognition in the mouse. J. Neurosci. 21, 8278–8285 11588199 10.1523/JNEUROSCI.21-20-08278.2001 PMC6763861

[B47] Ferguson J. N. Young L. J. Hearn E. F. Matzuk M. M. Insel T. R. Winslow J. T. (2000). Social amnesia in mice lacking the oxytocin gene. Nat. Genet. 25, 284–288 10.1038/77040 10888874

[B48] Fliessbach K. Weber B. Trautner P. Dohmen T. Sunde U. Elger C. E. (2007). Social comparison affects reward-related brain activity in the human ventral striatum. Science 318, 1305–1308 10.1126/science.1145876 18033886

[B49] Fone K. C. F. Porkess M. V. (2008). Behavioural and neurochemical effects of post-weaning social isolation in rodents - Relevance to developmental neuropsychiatric disorders. Neurosci. Biobehav. Rev. 32, 1087–1102 10.1016/j.neubiorev.2008.03.003 18423591

[B50] Forsythe R. Horowitz J. L. Savin N. E. Sefton M. (1994). Fairness in simple bargaining experiments. Games Econ. Behav. 6, 347–369 10.1006/game.1994.1021

[B51] Fouragnan E. Chierchia G. Greiner S. Neveu R. Avesani P. Coricelli G. (2013). Reputational priors magnify striatal responses to violations of trust. J. Neurosci. 33, 3602–3611 10.1523/JNEUROSCI.3086-12.2013 23426687 PMC6619519

[B52] Francis S. Rolls E. T. Bowtell R. Mcglone F. O'doherty J. Browning A. (1999). The representation of pleasant touch in the brain and its relationship with taste and olfactory areas. Neuroreport 10, 453–459 10.1097/00001756-199902250-00003 10208571

[B53] Freeman S. M. Smith A. L. Goodman M. M. Young L. J. (2012). *In vivo* and *in vitro* methods for localizing the oxytocin receptor in primate tissue. Am. J. Primatol. 74, 71–71

[B54] Fudge J. L. Kunishio K. Walsh P. Richard C. Haber S. N. (2002). Amygdaloid projections to ventromedial striatal subterritories in the primate. Neuroscience 110, 257–275 10.1016/S0306-4522(01)00546-2 11958868

[B55] Gerfen C. R. Engber T. M. Mahan L. C. Susel Z. Chase T. N. Monsma F. J. (1990). D1 and D2 dopamine receptor-regulated gene expression of striatonigral and striatopallidal neurons. Science 250, 1429–1432 10.1126/science.2147780 2147780

[B56] Geyer M. A. Wilkinson L. S. Humby T. Robbins T. W. (1993). Isolation rearing of rats produces a deficit in prepulse inhibition of acoustic startle similar to that in schizophrenia. Biol. Psychiatry 34, 361–372 10.1016/0006-3223(93)90180-L 8218603

[B57] Gibbons R. (1992). Game Theory for Applied Economists. Princeton, NJ: Princeton University Press

[B58] Gimpl G. Fahrenholz F. (2001). The oxytocin receptor system: structure, function, and regulation. Physiol. Rev. 81, 629–683 11274341 10.1152/physrev.2001.81.2.629

[B59] Glimcher P. (2010). Foundations of Neuroeconomic Analysis. New York, NY: Oxford University Press 10.1093/acprof:oso/9780199744251.001.0001

[B60] Goldstein B. L. Barnett B. R. Vasquez G. Tobia S. C. Kashtelyan V. Burton A. C. (2012). Ventral striatum encodes past and predicted value independent of motor contingencies. J. Neurosci. 32, 2027–2036 10.1523/JNEUROSCI.5349-11.2012 22323717 PMC3287081

[B61] Gross C. G. (1992). Representation of visual stimuli in inferior temporal cortex. Philos. Trans. Biol. Sci. 335, 3–10 10.1098/rstb.1992.0001 1348134

[B62] Güth W. Schmittberger R. Schwarze B. (1982). An experimental analysis of ultimatum bargaining. J. Econ. Behav. Organ. 3, 367–388 10.1016/0167-2681(82)90011-7

[B63] Haber S. N. (2003). The primate basal ganglia: parallel and integrative networks. J. Chem. Neuroanat. 26, 317–330 10.1016/j.jchemneu.2003.10.003 14729134

[B64] Haber S. N. Kim K. S. Mailly P. Calzavara R. (2006). Reward-related cortical inputs define a large striatal region in primates that interface with associative cortical connections, providing a substrate for incentive-based learning. J. Neurosci. 26, 8368–8376 10.1523/JNEUROSCI.0271-06.2006 16899732 PMC6673798

[B65] Haber S. N. Knutson B. (2010). The reward circuit: linking primate anatomy and human imaging. Neuropsychopharmacology 35, 4–26 10.1038/npp.2009.129 19812543 PMC3055449

[B66] Hall F. S. Wilkinson L. S. Humby T. Inglis W. Kendall D. A. Marsden C. A. (1998). Isolation rearing in rats: pre- and postsynaptic changes in striatal dopaminergic systems. Pharmacol. Biochem. Behav. 59, 859–872 10.1016/S0091-3057(97)00510-8 9586842

[B67] Hall F. S. Wilkinson L. S. Humby T. Robbins T. W. (1999). Maternal deprivation of neonatal rats produces enduring changes in dopamine function. Synapse 32, 37–43 10188636 10.1002/(SICI)1098-2396(199904)32:1<37::AID-SYN5>3.0.CO;2-4

[B68] Hammock E. A. D. Young L. J. (2006). Oxytocin, vasopressin and pair bonding: implications for autism. Philos. Trans. R. Soc. B Biol. Sci. 361, 2187–2198 10.1098/rstb.2006.1939 17118932 PMC1764849

[B69] Harbaugh W. T. Mayr U. Burghart D. R. (2007). Neural responses to taxation and voluntary giving reveal motives for charitable donations. Science 316, 1622–1625 10.1126/science.1140738 17569866

[B70] Herrnstein R. J. (1961). Relative and absolute strength of response as a function of frequency of reinforcement. J. Exp. Anal. Behav. 4, 267–272 10.1901/jeab.1961.4-267 13713775 PMC1404074

[B71] Hikosaka O. Sakamoto M. Usui S. (1989a). Functional properties of monkey caudate neurons I: activities related to saccadic eye movements. J. Neurophysiol. 61, 780–799 2723720 10.1152/jn.1989.61.4.780

[B72] Hikosaka O. Sakamoto M. Usui S. (1989b). Functional properties of monkey caudate neurons III: activites related to expectation of target and reward. J. Neurophysiol. 61, 814–833 2723722 10.1152/jn.1989.61.4.814

[B73] Hikosaka O. Takikawa Y. Kawagoe R. (2000). Role of the basal ganglia in the control of purposive saccadic eye movements. Physiol. Rev. 80, 953–978 10893428 10.1152/physrev.2000.80.3.953

[B74] Histed M. H. Pasupathy A. Miller E. K. (2009). Learning substrates in the primate prefrontal cortex and striatum: sustained activity related to successful actions. Neuron 63, 244–253 10.1016/j.neuron.2009.06.019 19640482 PMC2874751

[B75] Hollerman J. R. Tremblay L. Schultz W. (1998). Influence of reward expectation on behavior-related neuronal activity in primate striatum. J. Neurophysiol. 80, 947–963 9705481 10.1152/jn.1998.80.2.947

[B76] Hollerman J. R. Tremblay L. Schultz W. (2000). Involvement of basal ganglia and orbitofrontal cortex in goal-directed behavior. Prog. Brain Res. 126, 193–215 10.1016/S0079-6123(00)26015-9 11105648

[B77] Howes S. R. Dalley J. W. Morrison C. H. Robbins T. W. Everitt B. J. (2000). Leftward shift in the acquisition of cocaine self-administration in isolation-reared rats: relationship to extracellular levels of dopamine, serotonin and glutamate in the nucleus accumbens and amygdala-striatal FOS expression. Psychopharmacology (Berl.) 151, 55–63 10.1007/s002130000451 10958117

[B78] Hsu M. Anen C. Quartz S. R. (2008). The right and the good: distributive justice and neural encoding of equity and efficiency. Science 320, 1092–1095 10.1126/science.1153651 18467558

[B79] Inoue K. Burkett J. P. Young L. J. (2013). Neuroanatomical distribution of μ-opioid receptor mRNA and binding in monogamous prairie voles (*Microtus ochrogaster*) and non-monogamous meadow voles (*Microtus pennsylvanicus*). Neuroscience 244, 122–133 10.1016/j.neuroscience.2013.03.035 23537838 PMC4327842

[B80] Insel T. R. (2010). The challenge of translation in social neuroscience: a review of oxytocin, vasopressin, and affiliative behavior. Neuron 65, 768–779 10.1016/j.neuron.2010.03.005 20346754 PMC2847497

[B81] Insel T. R. Shapiro L. E. (1992). Oxytocin receptor distribution reflects social organization in monogamous and polygamous voles. Proc. Natl. Acad. Sci. U.S.A. 89, 5981–5985 10.1073/pnas.89.13.5981 1321430 PMC402122

[B82] Isovich E. Engelmann M. Landgraf R. Fuchs E. (2001). Social isolation after a single defeat reduces striatal dopamine transporter binding in rats. Eur. J. Neurosci. 13, 1254–1256 10.1046/j.0953-816x.2001.01492.x 11285023

[B83] Izuma K. (2012). The social neuroscience of reputation. Neurosci. Res. 72, 283–288 10.1016/j.neures.2012.01.003 22285602

[B84] Izuma K. Matsumoto K. Camerer C. F. Adolphs R. (2011). Insensitivity to social reputation in autism. Proc. Natl. Acad. Sci. U.S.A. 108, 17302–17307 10.1073/pnas.1107038108 21987799 PMC3198313

[B85] Izuma K. Saito D. N. Sadato N. (2008). Processing of social and monetary rewards in the human striatum. Neuron 58, 284–294 10.1016/j.neuron.2008.03.020 18439412

[B86] Kawagoe R. Takikawa Y. Hikosaka O. (1998). Expectation of reward modulates cognitive signals in the basal ganglia. Nat. Neurosci. 1, 411–416 10.1038/1625 10196532

[B87] Kawaguchi Y. Wilson C. J. Emson P. C. (1990). Projection subtypes of rat neostriatal matrix cells revealed by intracellular injection of biocytin. J. Neurosci. 10, 3421–3438 1698947 10.1523/JNEUROSCI.10-10-03421.1990 PMC6570194

[B88] Keverne E. B. Curley J. P. (2004). Vasopressin, oxytocin and social behaviour. Curr. Opin. Neurobiol. 14, 777–783 10.1016/j.conb.2004.10.006 15582383

[B89] Kimchi E. Y. Laubach M. (2009). Dynamic encoding of action selection by the medial striatum. J. Neurosci. 29, 3148–3159 10.1523/JNEUROSCI.5206-08.2009 19279252 PMC3415331

[B90] Kimchi E. Y. Torregrossa M. M. Taylor J. R. Laubach M. (2009). Neuronal correlates of instrumental learning in the dorsal striatum. J. Neurophysiol. 102, 475–489 10.1152/jn.00262.2009 19439679 PMC2712266

[B91] King-Casas B. Tomlin D. Anen C. Camerer C. F. Quartz S. R. Montague P. R. (2005). Getting to know you: reputation and trust in a two-person economic exchange. Science 308, 78–83 10.1126/science.1108062 15802598

[B92] Klein J. T. Deaner R. O. Platt M. L. (2008). Neural correlates of social target value in macaque parietal cortex. Curr. Biol. 18, 419–424 10.1016/j.cub.2008.02.047 18356054 PMC2362498

[B93] Klein J. T. Platt M. L. (2013). Social information signaling by neurons in primate striatum. Curr. Biol. 23, 691–696 10.1016/j.cub.2013.03.022 23562270 PMC3654103

[B94] Kobayashi S. Kawagoe R. Takikawa Y. Koizumi M. Sakagami M. Hikosaka O. (2006). Functional differences between macaque prefrontal cortex and caudate nucleus during eye movements with and without reward. Exp. Brain Res. 176, 341–355 10.1007/s00221-006-0622-4 16902776

[B95] Korn C. W. Prehn K. Park S. Q. Walter H. Heekeren H. R. (2012). Positively biased processing of self-relevant social feedback. J. Neurosci. 32, 16832–16844 10.1523/JNEUROSCI.3016-12.2012 23175836 PMC6621762

[B96] Kosfeld M. Heinrichs M. Zak P. J. Fischbacher U. Fehr E. (2005). Oxytocin increases trust in humans. Nature 435, 673–676 10.1038/nature03701 15931222

[B97] Künzle H. (1975). Bilateral projections from precentral motor cortex to the putamen and other parts of the basal ganglia. An autoradiographic study in *Macaca Fascicularis.* Brain Res. 88, 195–209 10.1016/0006-8993(75)90384-4 50112

[B98] Lau B. Glimcher P. W. (2007). Action and outcome encoding in the primate caudate nucleus. J. Neurosci. 27, 14502–14514 10.1523/JNEUROSCI.3060-07.2007 18160658 PMC6673449

[B99] Lau B. Glimcher P. W. (2008). Value representations in the primate striatum during matching behavior. Neuron 58, 451–463 10.1016/j.neuron.2008.02.021 18466754 PMC2427158

[B100] Laughlin S. B. Sejnowski T. J. (2003). Communication in neuronal networks. Science 301, 1870–1874 10.1126/science.1089662 14512617 PMC2930149

[B101] Lauwereyns J. Takikawa Y. Kawagoe R. Kobayashi S. Koizumi M. Coe B. (2002). Feature-based anticipation of cues that predict reward in monkey caudate nucleus. Neuron 33, 463–473 10.1016/S0896-6273(02)00571-8 11832232

[B102] Lim M. M. Murphy A. Z. Young L. J. (2004a). Ventral striatopallidal oxytocin and vasopressin V1a receptors in the monogamous prairie vole (*Microtus ochrogaster*). J. Comp. Neurol. 468, 555–570 10.1002/cne.10973 14689486

[B103] Lim M. M. Wang Z. Olazabal D. E. Ren X. Terwilliger E. F. Young L. J. (2004b). Enhanced partner preference in a promiscuous species by manipulating the expression of a single gene. Nature 429, 754–757 10.1038/nature02539 15201909

[B104] Loewenstein G. Thompson L. Bazerman M. H. (1989). Social utility and decision making in interpersonal contexts. J. Pers. Soc. Psychol. 57, 426–441 10.1037/0022-3514.57.3.426

[B105] Martin L. J. Spicer D. M. Lewis M. H. Gluck J. P. Cork L. C. (1991). Social deprivation of infant Rhesus monkeys alters the chemoarchitecture of the brain. 1. Subcortical regions. J. Neurosci. 11, 3344-3358 1682426 10.1523/JNEUROSCI.11-11-03344.1991 PMC6575550

[B106] Mobbs D. Yu R. Meyer M. Passamonti L. Seymour B. Calder A. J. (2009). A key role for similarity in vicarious reward. Science 324, 900–900 10.1126/science.1170539 19443777 PMC2839480

[B107] Mogenson G. J. Jones D. L. Yim C. Y. (1980). From motivation to action—functional interface between the limbic system and the motor system. Prog. Neurobiol. 14, 69–97 10.1016/0301-0082(80)90018-0 6999537

[B108] Moll J. Krueger F. Zahn R. Pardini M. De Oliveira-Souza R. Grafman J. (2006). Human fronto-mesolimbic networks guide decisions about charitable donation. Proc. Natl. Acad. Sci. U.S.A. 103, 15623–15628 10.1073/pnas.0604475103 17030808 PMC1622872

[B109] Morgan D. Grant K. A. Gage H. D. Mach R. H. Kaplan J. R. Prioleau O. (2002). Social dominance in monkeys: dopamine D2 receptors and cocaine self-administration. Nat. Neurosci. 5, 169–174 10.1038/nn798 11802171

[B110] Nader M. A. Morgan D. Gage H. D. Nader S. H. Calhoun T. L. Buchheimer N. (2006). PET imaging of dopamine D2 receptors during chronic cocaine self-administration in monkeys. Nat. Neurosci. 9, 1050–1056 10.1038/nn1737 16829955

[B111] Nowak M. A. (2006). Five rules for the evolution of cooperation. Science 314, 1560–1563 10.1126/science.1133755 17158317 PMC3279745

[B112] Olausson H. Lamarre Y. Backlund H. Morin C. Wallin B. G. Starck G. (2002). Unmyelinated tactile afferents signal touch and project to insular cortex. Nat. Neurosci. 5, 900–904 10.1038/nn896 12145636

[B113] O'Neill M. Schultz W. (2010). Coding of reward risk by orbitofrontal neurons is mostly distinct from coding of reward value. Neuron 68, 789–800 10.1016/j.neuron.2010.09.031 21092866

[B114] Oram M. W. Perrett D. (1996). Integration of form and motion in the anterior superior temporal polysensory area (STPa) of the macaque monkey. J. Neurophysiol. 76, 109–130 8836213 10.1152/jn.1996.76.1.109

[B115] Parent A. Bouchard C. Smith Y. (1984). The striatopallidal and striatonigral projections: two distinct fiber systems in primate. Brain Res. 303, 385–390 10.1016/0006-8993(84)91224-1 6744030

[B116] Parent A. Hazrati L. N. (1995). Functional anatomy of the basal ganglia: 1. The cortico-basal ganglia-thalamo-cortical loop. Brain Res. Rev. 20, 91–127 10.1016/0165-0173(94)00007-C 7711769

[B117] Perrett D. Smith P. A. J. Potter D. D. Mistlin A. J. Head A. S. Milner A. D. Jeeves M. A. (1984). Neurones responsive to faces in the temporal cortex: studies of funcional organization, sensitivity to identity and relation to perception. Hum. Neurobiol. 3, 197–208 6526706

[B118] Perrett D. Smith P. A. J. Potter D. D. Mistlin A. J. Head A. S. Milner A. D. Jeeves M. A. (1985a). Visual cells in the temporal cortex sensitive to face view and gaze direction. Proc. Biol. Sci. 223, 293–317 10.1098/rspb.1985.0003 2858100

[B119] Perrett D. I. Smith P. A. Mistlin A. J. Chitty A. J. Head A. S. Potter D. D. (1985b). Visual analysis of body movements by neurones in the temporal cortex of the macaque monkey: a preliminary report. Behav. Brain Res. 16, 153–170 10.1016/0166-4328(85)90089-0 4041214

[B120] Perrodin C. Kayser C. Logothetis N. K. Petkov C. I. (2011). Voice cells in the primate temporal lobe. Curr. Biol. 21, 1408–1415 10.1016/j.cub.2011.07.028 21835625 PMC3398143

[B121] Phan K. L. Sripada C. S. Angstadt M. Mccabe K. (2010). Reputation for reciprocity engages the brain reward center. Proc. Natl. Acad. Sci. U.S.A. 107, 13099–13104 10.1073/pnas.1008137107 20615982 PMC2919895

[B122] Powell S. B. Geyer M. A. Preece M. A. Pitcher L. K. Reynolds G. P. Swerdlow N. R. (2003). Dopamine depletion of the nucleus accumbens reverses isolation-induced deficits in prepulse inhibition in rats. Neuroscience 119, 233–240 10.1016/S0306-4522(03)00122-2 12763084

[B123] Rabin M. (1993). Incorporating fairness into game theory and economics. Am. Econ. Rev. 83, 1281–1302

[B124] Ravel S. Legallet E. Apicella P. (2003). Responses of tonically active neurons in the monkey striatum discriminate between motivationally opposing stimuli. J. Neurosci. 23, 8489–8497 13679417 10.1523/JNEUROSCI.23-24-08489.2003 PMC6740365

[B125] Resendez S. L. Dome M. Gormley G. Franco D. Nevarez N. Hamid A. A. (2013). mu-Opioid receptors within subregions of the striatum mediate pair bond formation through parallel yet distinct reward mechanisms. J. Neurosci. 33, 9140–9149 10.1523/JNEUROSCI.4123-12.2013 23699524 PMC6705037

[B126] Rilling J. Gutman D. Zeh T. Pagnoni G. Berns G. Kilts C. (2002). A neural basis for social cooperation. Neuron 35, 395–405 10.1016/S0896-6273(02)00755-9 12160756

[B127] Rodriguiz R. M. Chu R. Caron M. G. Wetsel W. C. (2004). Aberrant responses in social interaction of dopamine transporter knockout mice. Behav. Brain Res. 148, 185–198 10.1016/S0166-4328(03)00187-6 14684259

[B128] Rolls E. T. Grabenhorst F. Parris B. A. (2008). Warm pleasant feelings in the brain. Neuroimage 41, 1504–1513 10.1016/j.neuroimage.2008.03.005 18468458

[B129] Romo R. Scarnati E. Schultz W. (1992). Role of primate basal ganglia and frontal cortex in the internal generation of movements. II. Movement-related activity in the anterior striatum. Exp. Brain Res. 91, 385–395 10.1007/BF00227835 1483513

[B130] Ross H. E. Freeman S. M. Spiegel L. L. Ren X. Terwilliger E. F. Young L. J. (2009). Variation in oxytocin receptor density in the nucleus accumbens has differential effects on affiliative behaviors in monogamous and polygamous voles. J. Neurosci. 29, 1312–1318 10.1523/JNEUROSCI.5039-08.2009 19193878 PMC2768419

[B131] Russchen F. T. Bakst I. Amaral D. G. Price J. L. (1985). The amygdalostriatal projections in the monke*y.* An anterograde tracing study. Brain Res. 329, 241–257 10.1016/0006-8993(85)90530-X 3978445

[B132] Russo S. J. Nestler E. J. (2013). The brain reward circuitry in mood disorders. Nat. Rev. Neurosci. 14, 609–625 10.1038/nrn3381 23942470 PMC3867253

[B133] Sallet J. Mars R. B. Noonan M. P. Andersson J. L. O'reilly J. X. Jbabdi S. (2011). Social network size affects neural circuits in macaques. Science 334, 697–700 10.1126/science.1210027 22053054

[B134] Samejima K. Ueda Y. Doya K. Kimura M. (2005). Representation of action-specific reward values in the striatum. Science 310, 1337–1340 10.1126/science.1115270 16311337

[B135] Schorscher-Petcu A. Dupre A. Tribollet E. (2009). Distribution of vasopressin and oxytocin binding sites in the brain and upper spinal cord of the common marmoset. Neurosci. Lett. 461, 217–222 10.1016/j.neulet.2009.06.016 19539696

[B136] Schultz R. T. (2005). Developmental deficits in social perception in autism: the role of the amygdala and fusiform face area. Int. J. Dev. Neurosci. 23, 125–141 10.1016/j.ijdevneu.2004.12.012 15749240

[B137] Schultz W. (2000). Multiple reward signals in the brain. Nat. Rev. Neurosci. 1, 199–207 10.1038/35044563 11257908

[B138] Schultz W. (2004). Neural coding of basic reward terms of animal learning theory, game theory, microeconomics and behavioural ecology. Curr. Opin. Neurobiol. 14, 139–147 10.1016/j.conb.2004.03.017 15082317

[B139] Schultz W. Apicella P. Scarnati E. Ljungberg T. (1992). Neuronal activity in monkey ventral striatum related to the expectation of reward. J. Neurosci. 12, 4595–4610 1464759 10.1523/JNEUROSCI.12-12-04595.1992 PMC6575755

[B140] Schultz W. Dickinson A. (2000). Neuronal coding of prediction errors. Annu. Rev. Neurosci. 23, 473–500 10.1146/annurev.neuro.23.1.473 10845072

[B141] Schultz W. Romo R. (1988). Neuronal activity in the monkey striatum during the initiation of movements. Exp. Brain Res. 71, 431–436 10.1007/BF00247503 3169174

[B142] Schulz J. M. Reynolds J. N. J. (2013). Pause and rebound: sensory control of cholinergic signaling in the striatum. Trends Neurosci. 36, 41–50 10.1016/j.tins.2012.09.006 23073210

[B143] Selemon L. D. Goldman-Rakic P. S. (1985). Longitudinal topography and interdigitation of corticostriatal projections in the rhesus monkey. J. Neurosci. 5, 776–794 2983048 10.1523/JNEUROSCI.05-03-00776.1985 PMC6565017

[B144] Shepherd S. V. Deaner R. O. Platt M. L. (2006). Social status gates social attention in monkeys. Curr. Biol. 16, R119–R120 10.1016/j.cub.2006.02.013 16488858

[B145] Silva-Gomez A. B. Rojas D. Juarez I. Flores G. (2003). Decreased dendritic spine density on prefrontal cortical and hippocampal pyramidal neurons in postweaning social isolation rats. Brain Res. 983, 128–136 10.1016/S0006-8993(03)03042-7 12914973

[B146] Smeltzer M. D. Curtis J. T. Aragona B. J. Wang Z. X. (2006). Dopamine, oxytocin, and vasopressin receptor binding in the medial prefrontal cortex of monogamous and promiscuous voles. Neurosci. Lett. 394, 146–151 10.1016/j.neulet.2005.10.019 16289323

[B147] Stalnaker T. A. Calhoon G. G. Ogawa M. Roesch M. R. Schoenbaum G. (2012). Reward prediction error signaling in posterior dorsomedial striatum is action specific. J. Neurosci. 32, 10296–10305 10.1523/JNEUROSCI.0832-12.2012 22836263 PMC3419368

[B148] Sutton R. S. Barto A. G. (1998). Reinforcement Learning: An Introduction. Cambridge, MA: The MIT press

[B149] Tepper J. M. Bolam J. P. (2004). Functional diversity and specificity of neostriatal interneurons. Curr. Opin. Neurobiol. 14, 685–692 10.1016/j.conb.2004.10.003 15582369

[B150] Tidey J. W. Miczek K. A. (1996). Social defeat stress selectively alters mesocorticolimbic dopamine release: an *in vivo* microdialysis study. Brain Res. 721, 140–149 10.1016/0006-8993(96)00159-X 8793094

[B151] Tomlin D. Kayali M. A. King-Casas B. Anen C. Camerer C. F. Quartz S. R. (2006). Agent-specific responses in the cingulate cortex during economic exchanges. Science 312, 1047–1050 10.1126/science.1125596 16709783

[B152] Tricomi E. Rangel A. Camerer C. F. O'doherty J. P. (2010). Neural evidence for inequality-averse social preferences. Nature 463, 1089–1091 10.1038/nature08785 20182511

[B153] Tsao D. Y. Freiwald W. A. Tootell R. B. H. Livingstone M. S. (2006). A cortical region consisting entirely of face-selective cells. Science 311, 670–674 10.1126/science.1119983 16456083 PMC2678572

[B154] Vanhoesen G. W. Yeterian E. H. Lavizzomourey R. (1981). Widespread corticostriate projections from temporal cortex of the Rhesus-monkey. J. Comp. Neurol. 199, 205–219 10.1002/cne.901990205 7251940

[B155] Von Neumann J. Morgenstern O. (1947). The Theory of Games and Economic Behavior. Princeton, NJ: Princeton University Press

[B156] Wang Z. Yu G. Cascio C. Liu Y. Gingrich B. Insel T. R. (1999). Dopamine D2 receptor-mediated regulation of partner preferences in female prairie voles (*Microtus ochrogaster*): a mechanism for pair bonding? Behav. Neurosci. 113, 602–611 10.1037/0735-7044.113.3.602 10443786

[B157] Watson K. K. Platt M. L. (2012). Social signals in primate orbitofrontal cortex. Curr. Biol. 22, 2268–2273 10.1016/j.cub.2012.10.016 23122847 PMC3518589

[B158] Wilkinson L. S. Killcross S. S. Humby T. Hall F. S. Geyer M. A. Robbins T. W. (1994). Social isolation in the rat produces developmentally specific deficits in prepulse inhibition of the acoustic startle response without disrupting latent inhibition. Neuropsychopharmacology 10, 61–72 10.1038/npp.1994.8 8179795

[B159] Wilkinson R. G. Pickett K. (2010). The Spirit Level: Why Equality is Better for Everyone. London: Penguin Books

[B160] Wilson C. J. (1998). Basal ganglia, in The synaptic organization of the brain, ed Shepherd G. M. (New York, NY: Oxford University Press), 329–375

[B161] Wolpert D. M. Doya K. Kawato M. (2003). A unifying computational framework for motor control and social interaction. Philos. Trans. R. Soc. B Biol. Sci. 358, 593–602 10.1098/rstb.2002.1238 12689384 PMC1693134

[B162] Xiang T. Ray D. Lohrenz T. Dayan P. Montague P. R. (2012). Computational phenotyping of two-person interactions reveals differential neural response to depth-of-thought. PLoS Comput. Biol. 8:e1002841 10.1371/journal.pcbi.1002841 23300423 PMC3531325

[B163] Young L. J. Wang Z. (2004). The neurobiology of pair bonding. Nat. Neurosci. 7, 1048–1054 10.1038/nn1327 15452576

